# Retraction: TET1 exerts its tumor suppressor function by regulating autophagy in glioma cells

**DOI:** 10.1042/BSR-20160523_RET

**Published:** 2021-05-14

**Keywords:** autophagy, CRISPR/Caspase-9, glioma, methylation, TET1

This Retraction follows an Expression of Concern relating to this article previously published by Portland Press.

This article is being retracted from *Bioscience Reports* at the request of the Editor-in-Chief and the Editorial Board following receipt of a notification from a reader, alerting the Editorial Board to duplicated images in Figures 2F, 2I and 4B which are also published in *Biologics: Targets and Therapy* (10.2147/BTT.S134920). Images are also duplicated within the *Bioscience Reports* article, in Figures 2F and 4B.

The authors have been contacted in regards to the retraction, and disagree with the Journal's decision to retract the article; however, the Editorial Board stand by the decision to retract given the quantity and extent of the concerns with the figures.

